# Modulatory effect of *Mangifera indica* against carbon tetrachloride induced kidney damage in rats

**DOI:** 10.1515/intox-2015-0027

**Published:** 2015-12

**Authors:** Olufunsho Awodele, Adejuwon Adewale Adeneye, Sheriff Aboyade Aiyeola, Adokiye Senibo Benebo

**Affiliations:** 1 Department of Pharmacology, Therapeutics and Toxicology, Faculty of Basic Medical Science, College of Medicine, University of Lagos, Idi-Araba, Suruiere, Lagos State, Nigeria; 2 Department of Pharmacology, Faculty of Basic Medical Science, Lagos State University College of Medicine, Ikeja G.R.A., Lagos State, Nigeria; 3 Department of Pathology and Forensic Medicine, Faculty of Basic Medical Science, Lagos State University College of Medicine, Ikeja G.R.A., Lagos State, Nigeria

**Keywords:** *Mangifera indica*, stem bark aqueous extract, carbon tetrachloride, reno-modulation, antioxidant

## Abstract

There is little scientific evidence on the local use of *Mangifera indica* in kidney diseases. This study investigated the reno-modulatory roles of the aqueous stem bark extract of *Mangifera indica* (MIASE) against CCl_4_-induced renal damage. Rats were treated intragastrically with 125, 250 and 500 mg/kg/day MIASE for 7 days before and after the administration of CCl_4_ (3 ml/kg of 30% CCl_4_, i.p.). Serum levels of electrolytes (Na+, K+, Cl^−^, HCO3^−^), urea and creatinine were determined. Renal tissue reduced glutathione (GSH), malondialdehyde (MDA), catalase (CAT), superoxide (SOD) activities were also assessed. The histopathological changes in kidneys were determined using standard methods. In CCl_4_ treated rats the results showed significant (*p*<0.05) increases in serum Na+, K+, Cl^−^, urea and creatinine. CCl_4_ also caused significant (*p*<0.05) decreases in renal tissue SOD, CAT and GSH and significant (*p*<0.05) increases in MDA. The oral MIASE treatment (125-500 mg/kg) was found to significantly (*p*<0.05) attenuate the increase in serum electrolytes, urea and creatinine. Similarly, MIASE significantly (*p*<0.05) attenuated the decrease in SOD, CAT and GSH levels and correspondingly attenuated increases in MDA. *Mangifera indica* may present a great prospect for drug development in the management of kidney disease with lipid peroxidation as its etiology.

## Introduction

Kidney diseases occur in all age groups with incidence between 1.5 per million and 3.0 per million in children (Fogo, 2007). Among the causes of kidney diseases are congenital abnormalities of the kidney and urinary tract, focal segmental glomerulosclerosis, hemolytic uremic syndrome, immune complex diseases (Foreman & Chan, 1988), exposure to drugs and chemicals. One of our previous studies (Awodele *et al*., 2010) and several other studies have however underscored the significant role of oxidative stress and lipid peroxidation in kidney diseases (Reeder *et al*., 2002; Reeder *et al*., 2008). In addition to its role in renal diseases, lipid peroxidation has also been documented to be one of the major mechanisms in the toxicity of drugs and environmental agents (Awodele & Akintonwa, 2012).

Traditional medicinal plants have been largely used in developing countries to supplement orthodox medicines and the use of these herbal preparations have been supported by the World Health Organization (WHO), provided their non-toxicity was established (WHO, 1985). Apart from using medicinal plants for curative purposes, several studies have shown the potentials of some medicinal plants in preventing and protecting against some systemic diseases and organ damage. Kaur *et al*. (2010) showed the modulatory role of alizarin from *Rubia cordifolia* L. against genotoxicity of mutagens; Kitagishi *et al*. (2012) demonstrated the protection offered by medicinal herbs against cancer mediated via the activation of tumor suppressor and Kumar *et al*., 2011 highlighted the immunomodulatory effects of some traditional medicinal plants. The commonest modulatory mechanism of these medicinal plants is via expression of antioxidants and scavenging of free radicals.

The tree *Mangifera indica* (family: Anarcardiaceae) is among the most economically and culturally important tropical rainforest medicinal plants in Asia and Africa, especially due to its edible fruits. It is widely known as Mango. Studies have reported *Mangifera indica* fruit (mango) to possess anti-diabetic, antioxidant, anti-viral, cardiotonic, hypotensive, and antiinflammatory properties (Barreto *et al*., 2008). The stem bark of *Mangifera indica* has been reported to exert several pharmacological activities with antispas-modic, analgesic, antipyretic, anti-oxidant, anti-tumor, anti-viral, anti-diabetic, anti-bone resorption and immunomodulatory effects (Kumar *et al*., 2009). These findings are very encouraging and indicate that this herb should be studied more extensively to confirm the results and reveal other potential therapeutic effects. In African traditional medicine, in particular among Yoruba, Hausa and Igbo communities in Nigeria, various parts of *Mangifera indica* trees are used in the treatment of different human and veterinary diseases, including malaria (Ene *et al*., 2010), dysentery, cough, typhoid fever infection (Alo *et al*., 2012). It is also an anti-diuretic, anti-emetic and cardiac herb (Barreto *et al*., 2008). A preliminary ethno-botanical survey of its use conducted among traditional herbalists in Lagos metropolis (Southwest Nigeria) showed that hot and cold water infusion of *Mangifera indica* stem bark is highly valued in the local management of both liver and kidney diseases. Recently, the aqueous stem bark extract of *Mangifera indica* was reported to offer protection against CCl_4_-induced hepatotoxicity in Wistar rats (Adeneye *et al*., 2015). Nevertheless, there is a dearth of scientific investigation into the possible protective role of the aqueous stem bark extract of *Mangifera indica* against nephrotoxicity. Thus the presented explorative study was aimed at confirming or refuting the value of the folkloric use of water infusion of *Mangifera indica* stem bark in the local treatment of renal diseases. Thus the reno-modulatory roles of 125–500 mg/kg/day of the *Mangifera indica* stem bark aqueous extract were investigated in CCl_4_-induced nephrotoxicity in adult Wistar rats.

## Material and methods

The plant collection and identification, preparation of the plant extract, qualitative phytochemical analyses of aqueous stem bark extract of *Mangifera indica* (MIASE), acute oral toxicity test of MIASE using preliminary dose test of up and down procedure were carried out as previously reported by Adeneye *et al*. (2015).

### Experimental animals

The purchase of experimental animals, acclimatization, housing and feeding were done as documented in our previous study (Adeneye *et al*., 2015). The investigation conforms to the Guide for the Care and Use of Laboratory Animals published by the U. S. National Institutes of Health (NIH Publication No. 85-23, revised 1996) for studies involving experimental animals.

### Experimental design

Drug-induced renal toxicity models applied in conducting this study used 30% carbon tetrachloride dissolved in olive oil according to the modified method of Lu *et al*. (2002). The study was performed in two phases (chemopreventive and curative) with each phase involving 36 male Wistar rats. The rats in each model were grouped into six groups of six rats each – three control and three treatment groups (Adeneye *et al*., 2015).

### Induction of CCl_4_-induced nephrotoxicity and oral drug treatment in the chemopreventive model

In this model of chemically-induced nephrotoxicity, rats were randomly divided into 6 groups of 6 rats each so that the weight differences within and between groups did not exceed ±20% (Adeneye *et al*., 2015). The treatment protocols included: Group I (Control): 10 ml/kg of 0.9% normal saline; Group II: 10 ml/kg of 0.9% normal saline; Group III: 10 mg/kg of ascorbic acid; Group IV: 125 mg/kg of MIASE; Group V: 250 mg/kg of MIASE; Group VI: 500 mg/kg of MIASE.

The aforementioned oral treatments were applied for seven consecutive days and twenty-four hours after the last oral pretreatment with ascorbic acid and graded doses of MIASE, the rats in groups II-VI were treated with single intraperitoneal injection of 3 ml/kg of 30% CCl_4_ dissolved in olive oil. Ascorbic acid, being a known potent antioxidant and nephroprotectant, was used as standard reference drug. The treated rats were then sacrificed humanely forty-eight hours post-CCl_4_ treatment.

### Induction of CCl_4_-induced nephrotoxicity and oral drug treatment in the curative model

In this model of chemically-induced nephrotoxicity, the rats were also randomly divided into 6 groups of 6 rats each so that the weight differences within and between groups did not exceed ±20% (Adeneye *et al*., 2015). The treatment protocols included: Group I (Control) 1 ml/kg of 0.9% normal saline intraperitoneally; Group II: 3 ml/kg of 30% CCl_4_ *i.p* 1 hour before oral treatment with 1 ml/kg of 0.9% normal saline; Group III: 3 ml/kg of 30% CCl_4_ *i.p* 1 hour before oral treatment with 10 mg/kg ascorbic acid; Group IV: 3 ml/kg of 30% CCl_4_ *i.p* 1 hour before oral treatment with 125 mg/kg of MIASE; Group V: 3 ml/kg of 30% CCl_4_ *i.p* 1 hour before oral treatment with 250 mg/kg of MIASE; Group VI: 3 ml/kg of 30% CCl_4_ *i.p* 1 hour before oral treatment with 500 mg/kg of MIASE.

Each treatment lasted 7 days. Twenty-four hours after the last treatment on day 7, the rats were sacrificed humanely under diethyl ether anesthesia (Adeneye *et al*., 2015).

### Collection of blood samples and kidneys for renal tissue oxidative stress markers

The blood samples and kidneys for renal tissue oxidative stress markers were collected using the methods as described by Adeneye *et al*. (2015).

### Determination of kidney tissue antioxidant activities and lipid peroxidation

The methods described by Adeneye *et al*. (2015) were used to determine the superoxide dismutase, catalase, reduced glutathione and malondialdehyde activities.

### Determination of serum renal function parameters

#### Serum creatinine determination

An aliquot of 0.5 ml of serum sample was added to 3.5 ml of picric acid. The mixture was centrifuged for 5 minutes. 3 ml of the supernatant was taken and to this 0.2 ml of 4N NaOH was added. The mixture was incubated for 1 minute and the absorbance was read at 520 nm. The concentration of creatinine was determined.

#### Serum urea determination

0.1 ml of serum sample was added into a universal bottle containing 19.9 ml of distilled water and the suspension was well shaken. 1 ml of the suspension was transferred into a test tube and 1 ml of color reagent was added followed by 1 ml of acid reagent. The mixture was heated in boiling water for 20 minutes. It was then cooled and the absorbance was read at 520 nm against blank.

#### Serum electrolyte determination

Serum levels of sodium, potassium, chloride, calcium, bicarbonate and phosphate were determined using the ISE 6000 BYY SFRI spectrophotometer. When powered on, the machine carries out self-calibration for all parameters. When calibration is complete, the sample is placed into the probe and the tun button on the machine is pressed on the screen of the machine. The machine aspirates the sample and beeps with a screen display “remove sample”. The machine then processes the sample and displays the result of the test. The results of the test are printed out, showing all the required electrolyte levels, namely: sodium, potassium, chloride, bicarbonate, calcium and phosphate.

### Histopathology of the kidneys from treated rats

The remaining of the pair of kidneys harvested was gently but briskly rinsed in 0.9% normal saline and fixed in 10% formo-saline. The kidney histology was processed according to the methods described by Adeneye *et al*. (2015).

### Statistical analysis

Statistical analysis was performed using Graph Pad Prism (Graph Pad Software – Version 5.0. Graph Pad Software Inc., La Jolla, California, U.S.A.). Data were expressed as mean ± S.D. for body weights and relative kidney weights and mean ± S.E.M. for biochemical and hematological assays. The data were analyzed using the one-way ANOVA for comparison between the control and treated groups and post hoc test conducted using Newman-Keuls'-test. The level of statistical significance was considered at *p*<0.05, *p*<0.001 and *p*<0.0001.

## Results

### Plant extraction and phytochemical analysis of *Mangifera indica* aqueous stem bark extract

A yield of 15% was obtained. Alkaloids, tannins, cardiac glycosides, flavonoids, phlobatinnins, reducing sugars and saponins were contained in the extract as reported in our previous study (Adeneye *et al*., 2015).

### Preliminary limit dose test of the up-and-down procedure of the acute oral toxicity test of MIASE in Wistar rats

Table 1 shows that doses of up to 5 000 mg/kg of *MIASE* resulted in no mortality. However, behavioral toxicities such as body scratching, feed refusal, reduced locomotor activity, and watery stools were observed, as earlier reported in our study (Adeneye *et al*., 2015).

**Table 1:** Sequence and results of the limit dose test of MIASE in young female Wistar rats.

Test sequence	Dose (mg/kg)	Short-term result (48 h)	Long-term result (12 days)
01	5 000	Survival	Survival
02	5 000	Survival	Survival
03	5 000	Survival	Survival

Adeneye *et al*., 2015

### Effect of 125–500 mg/kg of MIASE on average body weight and relative organ weight in the chemopreventive model of CCl_4_-treated rats

Table 2 shows the effect of MIASE oral pretreatments on average body weight and relative organ weight of the kidney of CCl_4_-treated animals on days 1 and 7 of the experiment. Intraperitoneal treatment with CCl_4_ caused significant (*p*<0.0001) weight loss and non-significant weight reduction in the relative organ weight of the kidneys of CCl_4_-treated rats compared to control (Table 2). However, oral pretreatments with 125–500 mg/kg/day of MIASE and subsequent intraperitoneal treatment with CCl_4_ caused significant dose-dependent (*p*<0.05, *p*<0.001 and *p*<0.0001) further weight loss and non-significant alterations in the relative organ weights (kidneys) when compared with CCl_4_-treated (Group II) rats (Table 2).

**Table 2:** Effects of oral pre-treatments of 125–500 mg/kg of MIASE on body weight and relative kidney weight of CCl_4_-treated rats.

Average body weight (g) on			
Groups	Day 1	Day 8	Relative kidney weight
I	166.30±39.42	173.20±36.03	0.88±0.31
II	201.50±22.59	171.20±29.14^f^	0.69±0.23
III	190.70±29.97	150.50±29.04^f^	0.69±0.13
IV	200.20±24.70	184.00±29.31^d^	0.76±0.21
V	196.00±25.88	178.20±28.51^e^	0.74±0.20
VI	204.30±14.39	164.70±14.62^f^	0.76±0.25

d,e, and f represent significant decreases at *p*<0.05, *p*<0.001 and *p*<0.0001 when compared to respective Group II values.

Group I: Control; Group II: 0.9% normal saline + CCl_4_; Group III: ascorbic acid + CCl_4_; Group IV: 125MIASE + CCl_4_; Group V: 250MIASE + CCl_4_; Group VI: 500MIASE + CCl_4_

### Effect of 125–500 mg/kg of MIASE on renal function parameters in rats with CCl_4_-chemoprevention

CCl_4_ treatment caused significant (*p*<0.05, *p*<0.0001) increases in serum Na+, K+, Cl^−^, urea and creatinine while causing significant (*p*<0.05, *p*<0.001) decreases in serum HCO_3_^−^ levels compared to control rats (Table 3). However, oral pretreatments with 125–500 mg/kg of MIASE significantly (*p*<0.05, *p*<0.001) attenuated the increase in serum levels of Na+, K+, Cl^−^, urea and creatinine, while significantly (*p*<0.05, *p*<0.001) increasing the serum HCO_3_^−^ levels compared to CCl_4_-treated rats (Table 3).

**Table 3:** Effect of oral pretreatment with 125–500 mg/kg/day of MIASE on serum Na^+^, K^+^, Cl^–^, HCO_3_^–^, urea and creatinine in CCl_4_-treated rats

Groups	Na^+^	K^+^	Cl^−^	HCO_3_^−^	urea	creatinine
	(mmol/l)	(mmol/l)	(mmol/l)	(mmol/l)	(mmol/l)	(μmol/l)
I	146.5±0.76	11.38±0.97	113.90±1.65	18.63±1.31	11.23±0.53	58.92±3.86
II	194.80±0.71^c^	7.55±0.52^d−^	142.40±1.01^c^	11.30±0.41^f−^	18.61±0.41^c^	93.37±2.80
III	142.90±1.30^f^	11.58±0.42	105.70±1.48^f^	17.43±0.68^c+^	8.33±0.39^f^	9.42±4.47^f^
IV	162.50±1.10^e^	13.97±1.79^a+^	126.80±4.49^d^	13.22±0.94^e−^	15.48±1.39	65.10±3.65^f^
V	143.20±0.56^f^	10.95±0.50	121.00±1.88	14.88±1.41^e^	11.13±1.00^d^	63.78±3.65^f^
VI	138.70±3.68^f^	12.38±0.95^a+^	107.30±1.74^f^	17.23±1.17^f^	8.03±0.75^d^	61.43±4.17^f^

c,d–,f– ^c^ represents a significant increase at *p*<0.0001 and ^d–^ and ^f–^ represent significant decreases at *p*<0.05 and *p*<0.0001, respectively, when compared to Group I values.

d,e and f represent significant decreases at *p*<0.05, *p*<0.001 and *p*<0.0001, respectively, when compared to Group II values. Group I: Control; Group II: 0.9% normal saline + CCl_4_; Group III: ascorbic acid + CCl_4_; Group IV: 125MIASE + CCl_4_; Group V: 250MIASE + CCl_4_; Group VI: 500MIASE + CCl_4_

### Effect of 125–500 mg/kg MIASE on renal tissue antioxidant status in CCl_4_ treated rats

Table 4 shows the effects of oral pre-treatments with 125–500 mg/kg/day of MIASE and subsequent intraperitoneal CCl_4_ treatment on antioxidant markers (SOD, CAT, GSH and MDA) in the treated rats. CCl_4_ treatment caused significant (*p*<0.05 and *p*<0.001) decreases in renal tissue SOD, CAT and GSH while causing significant (*p*<0.001 and *p*<0.0001) increases in renal tissue MDA (Table 4). Oral pretreatment with 250 and 500 mg/kg of MIASE significantly (*p*<0.05 and *p*<0.0001) attenuated decreases in renal tissue levels of SOD, CAT and GSH levels while it significantly attenuating increases in renal tissue MDA levels (Table 4). These results were comparable to the effect recorded for the 10 mg/kg of the standard antioxidant (ascorbic acid) used. However, 125 mg/kg/day of MIASE did cause significant alterations in the renal tissue levels of SOD, CAT, GSH and MDA when compared to the effect in the CCl_4_-treated rats of Group II (Table 4).

**Table 4:** Effect of oral pretreatment with 125–500 mg/kg/day of MIASE on renal tissue SOD, CAT, GSH and MDA in the CCl_4_-treated rats.

Group	SOD (U/mg protein)	CAT (U/mg protein)	GSH (U/mg protein)	MDA (U/mg protein)
I	5.16±0.58	20.17±1.94	2.90±0.35	0.11±0.05
II	2.92±0.35^a−^	16.25±0.67^a−^	1.44±0.19^b−^	0.59±0.13^c^
III	6.16±0.31^c+^	23.18±1.70^a+^	3.09±0.13^c+^	0.06±0.02^f^
IV	3.67±0.68	18.01±2.84	1.80±0.22	0.18±0.03^e^
V	4.18±0.38^a+^	22.54±0.86^a+^	3.05±0.27^c+^	0.08±0.02^f^
VI	5.24±0.43^a+^	27.95±2.40^c+^	4.49±0.32^c+^	0.04±0.01^f^

a−,b−,a+,c+ ^a−^ and ^b−^ represent significant decreases at *p*<0.05 and *p*<0.001, respectively, when compared to Group I values while ^a+^, and ^c+^ represent significant increases at *p*<0.05 and *p*<0.0001, respectively, when compared to Group II values.

c,e,f ^c^ represents a significant increase at *p*<0.0001 when compared to Group II values while ^e^ and ^f^ represent significant decreases at *p*<0.001 and *p*<0.0001, respectively, when compared to Group II values.

Group I: Control; Group II: 0.9% normal saline + CCl_4_; Group III: ascorbic acid + CCl_4_; Group IV: 125MIASE + CCl_4_; Group V: 250MIASE + CCl_4_; Group VI: 500MIASE + CCl_4_

### Histopathological results of oral pretreatment with 125–500 mg/kg MIASE on kidneys of CCl_4_-treated rats

**Figure 1:**
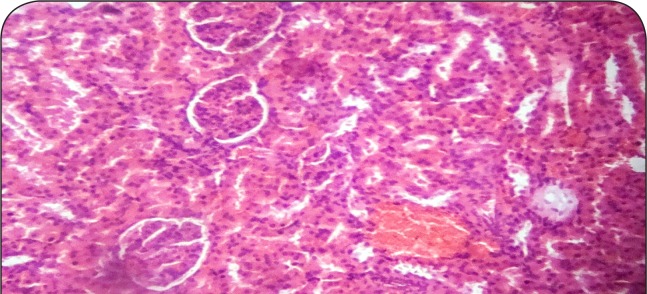
Sectional representation of normal rat kidney showing normal glomeruli and tubules with a localized single area of peritubular hemorrhage (hematoxylin & eosin stain, x100 magnification).

**Figure 2:**
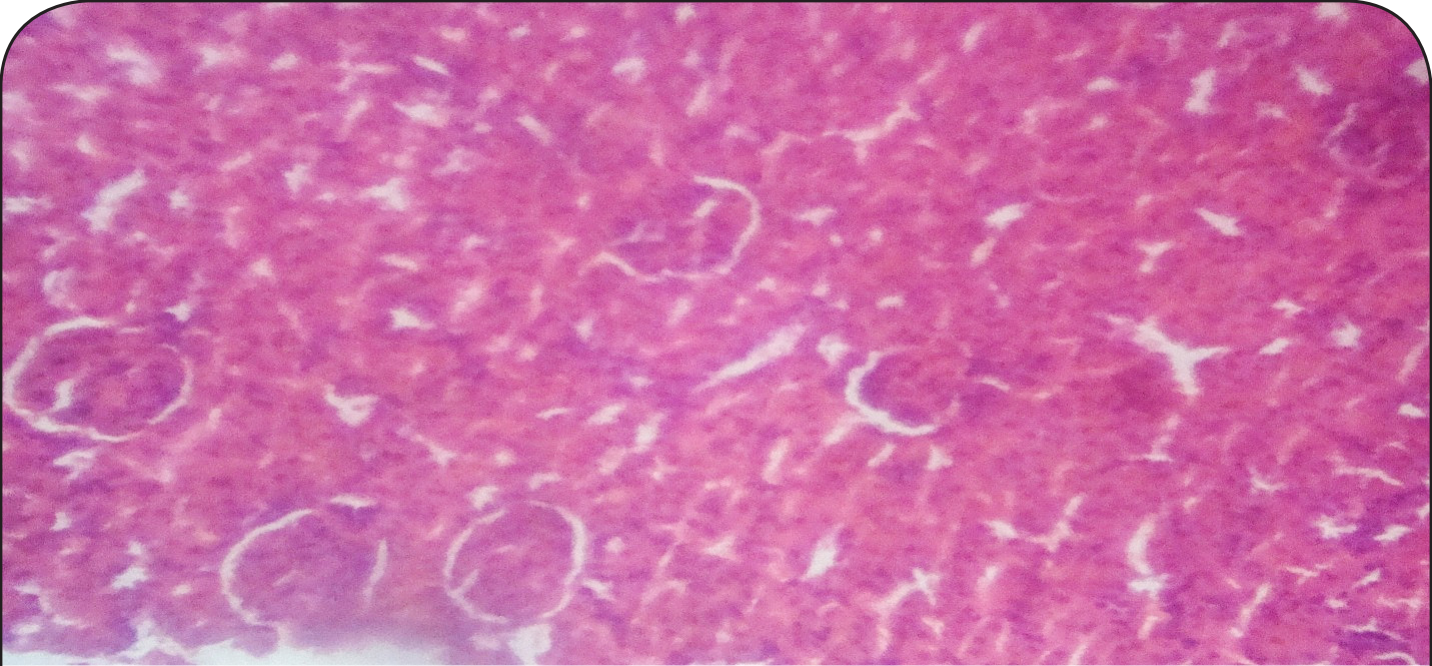
Sectional representation of CCl4-treated rat kidney showing focal glomerular atrophy and tubular congestion and necrosis (hematoxylin & eosin, ×400 magnification).

**Figure 3:**
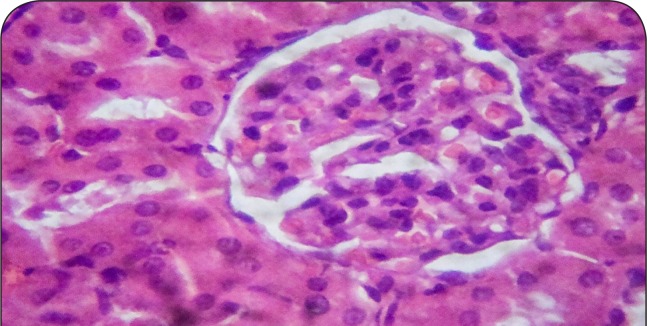
Sectional representation of CCl_4_-treated rat kidney pretreated with 10 mg/kg/day of vitamin C showing normal glomerulus and mild tubular congestion (hematoxylin & eosin, ×400 magnification).

**Figure 4:**
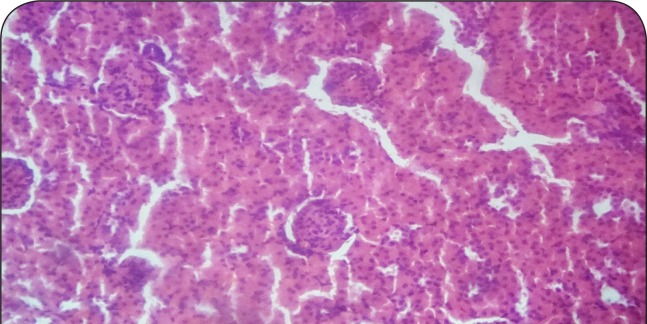
Sectional representation of CCl_4_-treated rat kidney pretreated with 125 mg/kg/day of MIASE showing intact glomeruli and moderate tubular necrosis (hematoxylin & eosin, x100 magnification).

**Figure 5:**
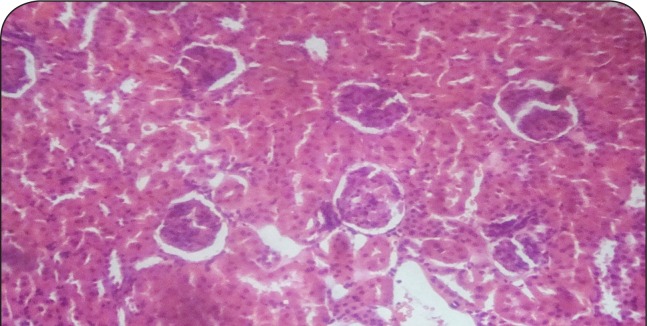
Sectional representation of CCl_4_-treated rat kidney pretreated with 250 mg/kg/day of MIASE showing intact glomeruli and moderate tubular congestion (hematoxylin & eosin, x100 magnification).

**Figure 6:**
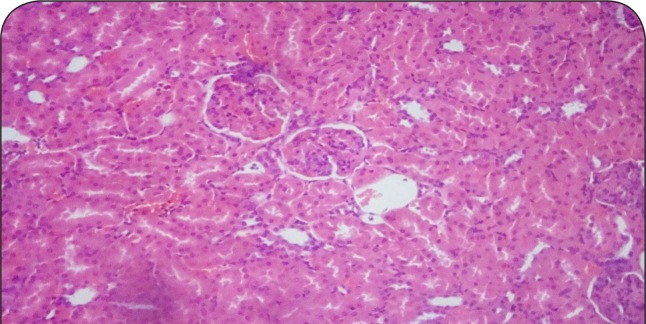
Sectional representation of CCl_4_-treated rat kidney pretreated with 500 mg/kg/day of MIASE showing intact glomeruli and mild tubular congestion (hematoxylin & eosin, ×100 magnification).

Figures 1–6 show the histopathological findings of oral pretreatments with 125–500 mg/kg/day of MIASE on the renal tissue of CCl_4_-treated rats. Single intraperitoneal treatment with CCl_4_ caused glomerular atrophy with tubular swelling and necrosis (Figure 2) compared to normal renal architecture (Figure 1). With repeated daily oral pretreatments with 10 mg/kg of ascorbic acid and 125–500 mg/kg/day of MIASE, these histological changes were ameliorated and improved in a dose-related manner (Figure 3–6).

### Effect of 125–500 mg/kg of MIASE on average body weight and relative kidney weights in the chemocurative model of CCl_4_-treated rats

Table 5 shows the effect of MIASE oral pretreatments on the average body weight and relative organ weight of the kidney of CCl_4_-treated, days 1 and 7 of the experiment. Intraperitoneal treatment with CCl_4_ caused significant (*p*<0.0001) weight loss changes and non-significant increase in the kidney relative weight of CCl_4_-treated rats compared to control rats (Table 5). However subsequent oral treatments with 125–500 mg/kg/day of MIASE after intraperitoneal treatment with CCl_4_ caused further significant dose-dependent weight loss (*p*<0.05, *p*<0.001 and *p*<0.0001) and non-significant alterations in the kidneys compared with CCl_4_-treated rats (Group II) (Table 5).

**Table 5:** Effect of 125–500 mg/kg of MIASE on average body weight and relative kidney weight in the chemocurative model of CCl4-treated rats.

Groups	Average body weight (g) on		%Δ	Relative kidney weight
	Day 1	Day 8		
I	149.00±19.18	190.20±20.17	27.96±4.07	0.64±0.02
II	170.30±24.75	196.30±20.94	14.28±4.80^c−^	0.73±0.03
III	174.50±18.68	216.70±25.91	24.04±3.42^b+^	0.65±0.03
IV	175.80±28.00	206.70±5.04	17.31±3.28^c−^	0.70±0.03
V	144.20±4.67	179.20±5.04	24.46±6.77^b+^	0.74±0.02
VI	149.30±12.31	194.60±19.05	27.64±2.99^c+^	0.67±0.02

c,c−,b+,c+ ^c^ and ^c−^ represent significant increase and decrease at *p*<0.0001, respectively, when compared to Group I values, while ^b+^ and ^c+^ represent significant increases at *p*<0.001 and *p*<0.0001, respectively, when compared to Group II values.

Group I: Control; Group II: 0.9% normal saline + CCl_4_; Group III: ascorbic acid + CCl_4_; Group IV: 125MIASE + CCl_4_; Group V: 250MIASE + CCl_4_; Group VI: 500MIASE + CCl_4_

### Effect of 125–500 mg/kg of MIASE on renal function parameters in CCl_4_-chemocurative rats

CCl_4_ treatment caused significant (*p*<0.05, *p*<0.0001) increases in serum Na+, K+, Cl^−^, urea and creatinine, while causing significant (*p*<0.05, *p*<0.001) decreases in serum HCO_3_^−^ levels compared to control rats (Table 6). However, post-CCl_4_ oral treatments with 125–500 mg/kg of MIASE significantly (*p*<0.001, *p*<0.0001) attenuated the increase in the serum levels of Na^+^, K^+^, Cl^−^, urea and creatinine dose-dependently, while significantly (*p*<0.0001) increasing the serum HCO_3_^−^ levels at 500 mg/kg of MIASE compared to the CCl_4_-treated rats (Table 6).

**Table 6:** Effect of 125–500 mg/kg of MIASE on renal function parameters in CCl4-chemocurative rats.

Groups	Na^+^ (mmol/l)	K^+^ (mmol/l)	Cl^–^ (mmol/l)	HCO3^–^ (mmol/l)	urea (mmol/l)	creatinine (μmol/l)
I	143.0±1.09	11.15±0.55	100.10±0.45	17.85±1.00	8.00±0.36	58.92±3.86
II	172.20±1.59^c^	17.45±1.17^c^	127.00±1.06^c^	11.50±0.39^f–^	18.22±0.07^c^	93.37±2.80
III	142.90±0.60^f^	9.80±0.15	106.10±0.46^f^	16.70±0.76^c+^	11.34±0.40^f^	59.42±4.47^f^
IV	157.20±2.67^e^	11.10±0.17^e^	116.90±0.59^e^	12.12±0.35	17.34±0.30^c^	65.10±3.65^f^
V	136.40±3.52^f^	11.07±0.44^e^	103.80±0.66^f^	12.72±0.45	10.43±0.79^f^	63.78±3.65^f^
VI	130.90±0.46^f^	9.59±0.15^f^	100.40±1.86^f^	16.72±0.44^c+^	9.33±0.22^f^	61.43±4.17^f^

c,f– represents a significant increase at *p*<0.0001 and ^f–^ represents significant decreases at *p*<0.0001 when compared to Group I values.

d,e,f,c+ ^d^, ^e^ and ^f^ represent significant decreases at *p*<0.05, *p*<0.001 and *p*<0.0001, respectively, while ^c+^ represents a significant increase at *p*<0.0001 when compared to Group II values.

Group I: Control; Group II: 0.9% normal saline + CCl_4_; Group III: ascorbic acid + CCl^4^; Group IV: 125MIASE + CCl_4_; Group V: 250MIASE + CCl_4_; Group VI: 500MIASE + CCl_4_

### Effect of 125–500 mg/kg MIASE on renal tissue antioxidant markers in CCl_4_-chemocurative rats

Table 7 shows the effects of 125–500 mg/kg of MIASE on renal tissue antioxidant markers (SOD, CAT, GSH and MDA) in the post-CT treated rats. CCl_4_ treatment caused significant (*p*<0.05 and *p*<0.0001) decreases of renal SOD, CAT and GSH while causing significant (*p*<0.001 and *p*<0.0001) increases in renal MDA values (Table 7). However, post-CCl_4_ oral treatments with 125500 mg/kg/day of MIASE significantly (*p*<0.05, *p*<0.001 and *p*<0.0001) reversed and improved the values of these markers when compared to values for the CCl_4_-treated rats, returning them to near normal values (Table 7).

**Table 7:** Effect of 125–500 mg/kg of MIASE on renal tissue antioxidant markers in CCl4-chemocurative in rats.

Group	SOD (U/mg protein)	CAT (U/mg protein)	GSH (U/mg protein)	MDA (U/mg protein)
I	3.35±0.24	27.42±2.11	0.56±0.05	0.08±0.01
II	2.14±0.17^c−^	13.20±0.53^c−^	0.37±0.03^a−^	0.11±0.01^c^
III	2.96±0.13^b+^	20.70±1.13^b+^	0.57±0.02^a+^	0.02±0.01^f^
IV	3.13±0.15^b+^	22.53±1.10^b+^	0.96±0.11^b+^	0.07±0.01^f^
V	4.07±0.16^c+^	26.11±1.06^c+^	1.16±0.14^c+^	0.01±0.00^f^
VI	4.22±0.23^c+^	27.70±1.58^c+^	1.35±0.15^c+^	0.01±0.00^f^

a−,c− ^a−^ and ^c−^ represent significant decreases at *p*<0.05 and *p*<0.0001, respectively, when compared to Group I values

a+,b+,c+ while ^a+,b+^ and ^c+^ represent significant increases at *p*<0.05, *p*<0.001 and *p*<0.0001, respectively, when compared to Group II values.

c,f ^c^ represents a significant increase at *p*<0.0001 when compared to Group II values, while ^f^ represents a significant decrease at *p*<0.0001 when compared to Group II values.

Group I: Control; Group II: 0.9% normal saline + CCl_4_; Group III: ascorbic acid + CCl_4_; Group IV: 125MIASE + CCl_4_; Group V: 250MIASE + CCl_4_; Group VI: 500MIASE + CCl_4_

### Histopathological results of post-CCl_4_ oral treatment with 125–500 mg/kg of MIASE on the renal tissue of CCl_4_-treated rats

**Figure 7:**
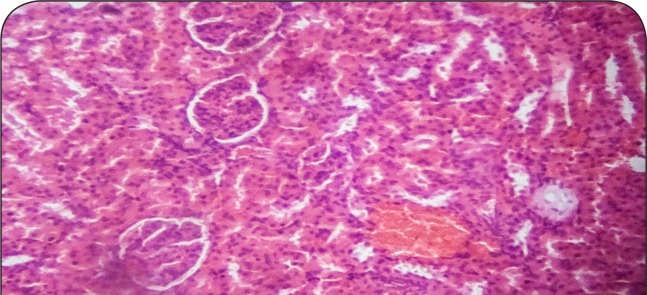
Sectional representation of normal rat kidney showing normal glomeruli and tubules with a localized single area of peritubular hemorrhage (hematoxylin & eosin stain, ×100 magnification).

**Figure 8:**
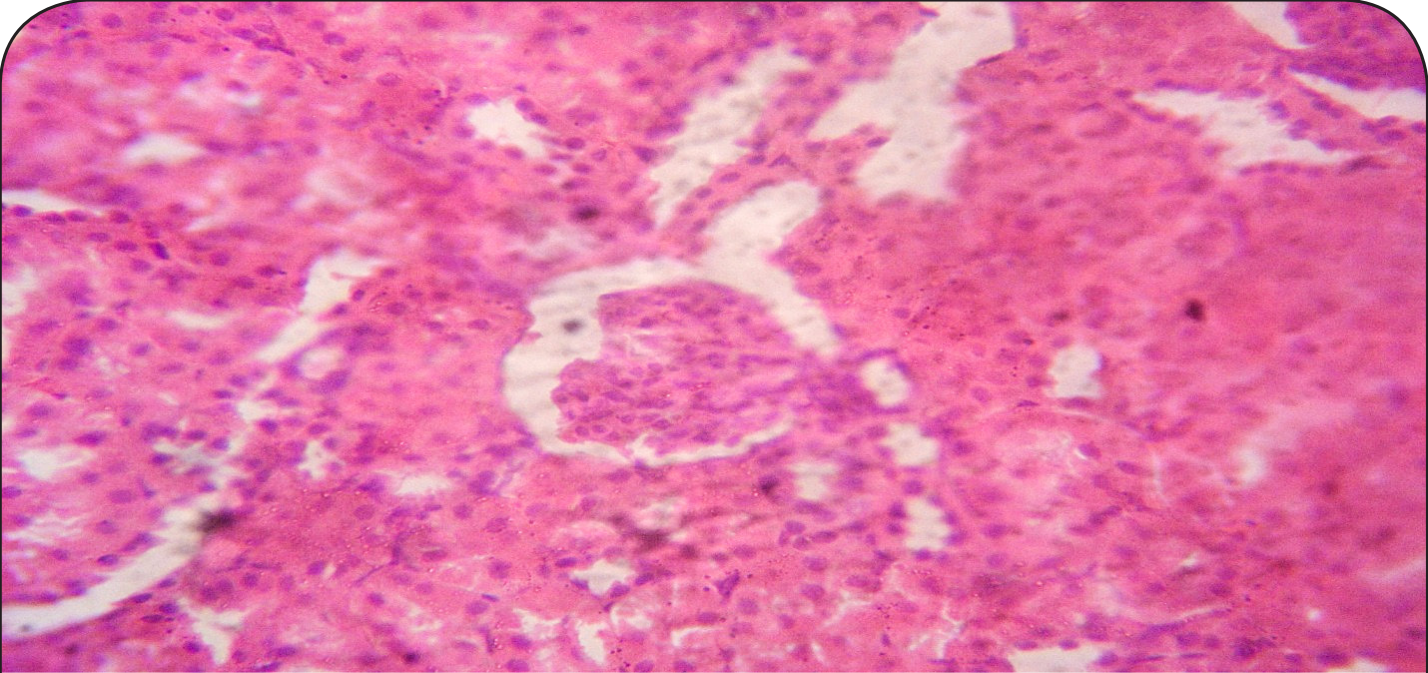
A representative section of CCl_4_ treated rat kidney showing severe tubular swellings and tubular lumen obliterations as well as tubular necrosis (hematoxylin & eosin, ×100 magnification).

**Figure 9:**
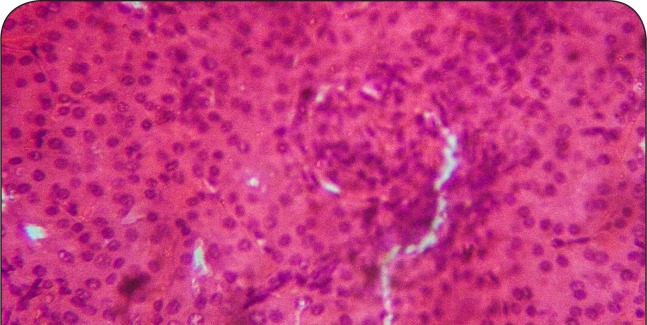
A representative section of post-CCl_4_ treated rat kidney treated with 10 mg/kg of vitamin C showing glomerular capillary congestion and mild tubular swelling (hematoxylin & eosin, ×100 magnification).

**Figure 10:**
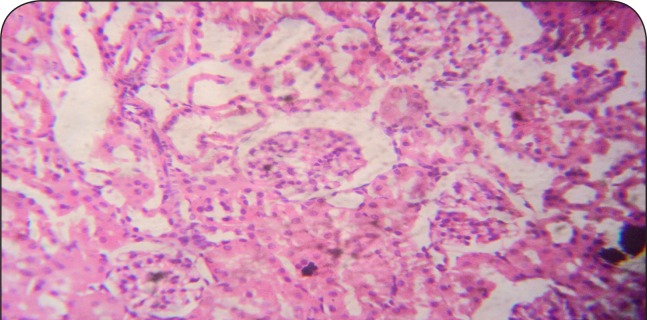
A representative section of post-CCl_4_ treated rat kidney treated with 125 mg/kg of MIASE showing severe tubular swellings and congestion (hematoxylin & eosin, ×100 magnification).

**Figure 11:**
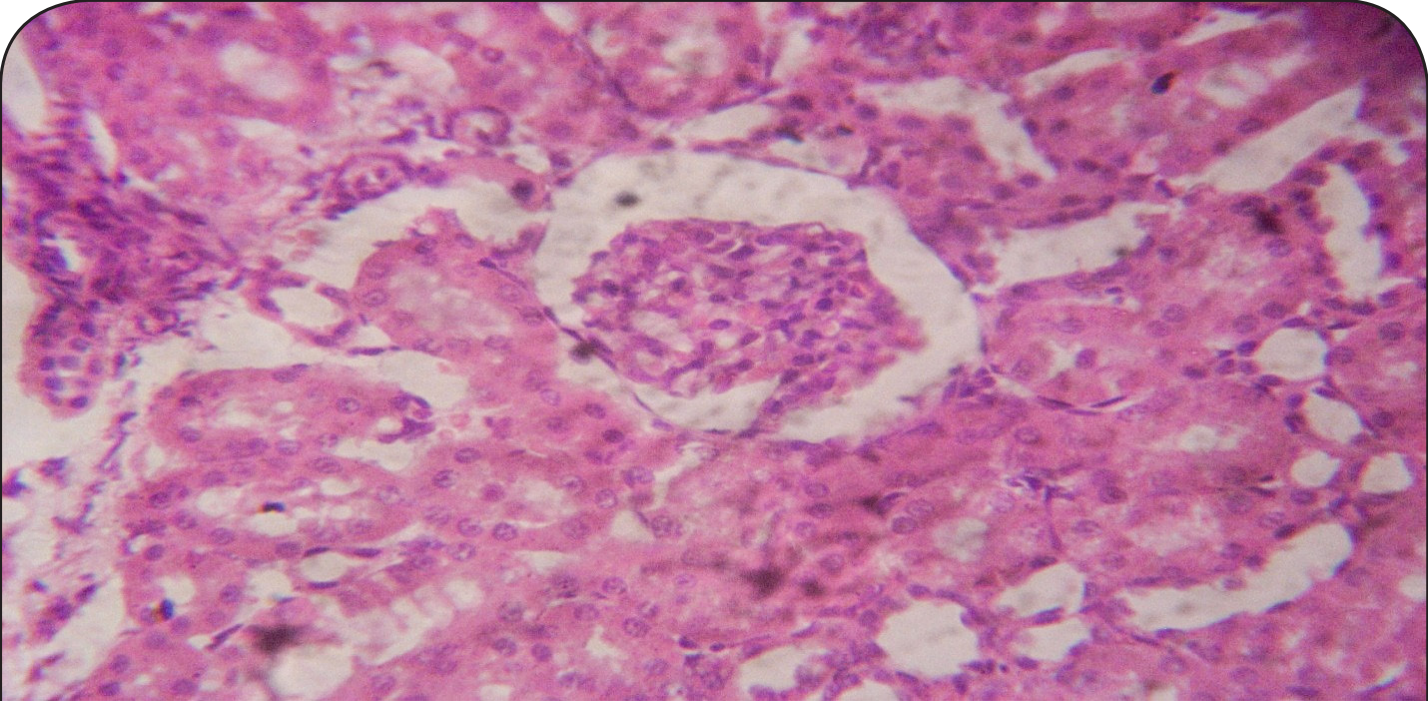
A representative section of post-CCl_4_ treated rat kidney treated with 250 mg/kg of MIASE showing mild-to-moderate tubular swellings and congestion with an intact glomerulus (hematoxylin & eosin, ×400 magnification).

**Figure 12:**
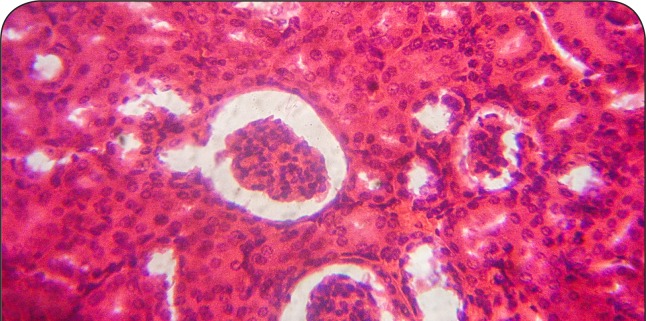
A representative section of post-CCl_4_ treated rat kidney treated with 500 mg/kg of MIASE showing mild tubular swellings and congestion with intact glomeruli (hematoxylin & eosin, ×400 magnification).

Intraperitoneal CCl_4_ treatment was associated with severe tubular swellings, tubular lumen obliterations and tubular necrosis (Figure 8) when compared to normal renal architecture (Figure 7). With repeated post-CCl_4_ oral treatment with 10 mg/kg/day of vitamin C and 125–500 mg/kg/day of MIASE, there was a dose related amelioration in the CCl_4_-induced renal lesions (Figures 9–12).

## Discussion

Exposure to carbon tetrachloride is on the increase due to environmental pollution. The exposure can come from the air, drinking water, foodstuffs and soil (ATSDR, 2005; IPCS, 1999). It could also be from certain industrial sites where carbon tetrachloride is still used or where previously industrial contamination had occurred (ATSDR, 2005). The liver and kidney are the major target organs for toxicity following acute inhalation or ingestion exposure to carbon tetrachloride (IPCS, 1998;1999). Liver damage can occur after 24 hours and in serious cases this can result in painful swollen liver, ascites, hemorrhages, hepatic coma and death (ATSDR, 2005; IPCS, 1999). Kidney damage with impairment in function normally occurs 2–3 weeks after exposure (IPCS, 1999), but in severe cases this can develop within 1–6 days in association with liver failure (ATSDR, 2005). Due to the fatality of kidney damage in affecting optimal human functions, attention should concentrate on strategies preventing the occurrence of kidney disease more than on palliative management.

In the recent past, several research studies on preventive strategies of renal damage have been conducted. Mesery *et al*. (2009) demonstrated the chemopreventive and renal protective effects of ocosahexaenoic acid (DHA); Pracheta *et al*, (2012) showed the chemopreventive effect of hydroethanolic extract of *Euphorbia neriifolia* leaves against DENA-induced renal carcinogenesis in mice and Sharma & Janmeda (2012) documented the chemopreventive role of *Euphorbia neriifolia* (Linn) and its isolated flavonoid against N-nitrosodiethylamine-induced renal histopathological damage in male mice.

A preliminary ethno-botanical use survey conducted among traditional herbalists in Lagos metropolis (Southwest Nigeria) showed that hot and cold water infusion of *Mangifera indica* stem bark is highly valued in the local management of both liver and kidney diseases. However, this assertion has not been scientifically investigated. Thus the present study investigated the reno-modulatory roles of the aqueous stem bark extract of *Mangifera indica* against CCl_4_ induced renal damage using rodent models.

The result of acute oral toxicity (LD_50_) study of aqueous stem bark extract of *Mangifera indica* showed no mortality at the maximum dose of 5000 mg/kg/body weight (Adeneye *et al*., 2015). In an acute oral toxicity study by Ogbe *et al*. (2012), *Mangifera indica* was documented to be non-lethal in animals at doses of 5000 mg/kg body weight. These results may indicate that the aqueous leaf extract of *Mangifera indica* is safe (non-lethal) during acute oral administration. The dose of 2 g/kg was reported as the ceiling point for medicinal plant toxicity when administered orally in acute toxicity studies (Lu *et al*., 1965; Awodele *et al*., 2012). But this safety affirmation is not applicable to long-term intake of medicinal plants. Behavioral toxicities manifested by the treated rats included body scratching, feed refusal, reduced locomotor activity, and watery stools.

Exposure of rodents to CCl_4_ in the present study showed significant (*p*<0.05, *p*<0.0001) increases in serum Na+, K+, Cl^−^, urea and creatinine, while causing significant (*p*<0.05, *p*<0.001) decreases in serum HCO_3_^−^ levels. These findings corroborate the previous data on CCl_4_ induction of renal damage (ATSDR, 2005; IPCS, 1999). The consistent damage of CCl_4_ on renal tissue may call for critical strategies to preserve/protect the kidneys of people who are occupationally or otherwise exposed to CCl_4_. MIASE (125-500 mg/kg), as shown in this study, significantly (*p*<0.05, *p*<0.001) attenuated the increase in the serum levels of Na^+^, K^+^, Cl^−^, urea and creatinine, while significantly (*p*<0.05, *p*<0.001) increasing serum HCO_3_^−^ levels. The results of histology of rat kidney tissues also revealed glomerular atrophy with tubular swelling and necrosis caused by CCl_4_. MIASE at oral doses of 250 and 500 mg/kg protected the rat kidney tissues from gross architectural alterations and damage. The chemopreventive potentials of MIASE (125–500 mg/kg) as shown in this study may scientifically affirm the use of *Mangifera indica* in kidney diseases in folk medicine. The speculated mechanism of kidney damage by CCl_4_ may occur via oxidative stress and lipid peroxidation, as shown by significant (*p*<0.05 and *p*<0.001) decreases in renal tissue SOD, CAT and GSH and significant (*p*<0.001 and *p*<0.0001) increases in renal tissue MDA. It may also be considered that MIASE (125–500 mg/kg) offered chemoprevention against kidney damage by enhancing the endogenous antioxidant activity in the treated rats, as implied in the significantly reduced (*p*<0.05 and *p*<0.0001) decreases in renal tissue levels of SOD, CAT and GSH and the attenuated increases in renal tissue MDA levels. It is also interesting to know that MIASE (125–500 mg/kg) demonstrated dose dependent chemocurative effects against CCl_4_-induced renal damage, with the highest reversal of kidney damage activity at 500 mg/kg.

On balance then, *Mangifera indica* possesses some antioxidant agents (Pardo-Andreu *et al*., 2006). The antioxidant properties of *Mangifera indica* could be attributed to its constituent flavonoids and other poly-phenolics as these phytocomponents have been widely reported to possess antioxidant activities (Roy *et al*., 2005; Yang *et al*., 2010). It is thus possible to speculate that the antioxidant properties of this plant are responsible for its reno-modulatory activities, as documented in this study. Antioxidants preventing or reversing kidney damage had been reported in our earlier study where vitamins C and E were found to protect against renal and testicular damage caused by *Alstonia boonei* (Awodele *et al*., 2010).

Based on the findings obtained in this study, it can be stated that *Mangifera indica* has the potential to prevent or reverse kidney damage. This readily available plant may offer great prospect for drug development in the management of acute renal disease, especially kidney disease with the etiology of oxidative stress and lipid peroxidation.
